# Usage of gloves for hair shampooing in German hairdressing salons

**DOI:** 10.1186/s12995-015-0089-y

**Published:** 2015-12-30

**Authors:** Madeleine Dulon, Björn Kähler, Sandra Kirvel, Günter Schlanstedt, Albert Nienhaus

**Affiliations:** Department of Occupational Health Research, German Social Accident Insurance Institution for the Health and Welfare Services, Pappelallee 33/35/37, 22089 Hamburg, Germany; Society for Research and Consulting in the Health and Welfare Sector, Prälat-Otto-Müller-Platz 2, 50670 Cologne, Germany; Institute for Health Services Research in Dermatology and Nursing (IVDP; CVcare), University Medical Centre Hamburg-Eppendorf, Martinistrasse 52, 20246 Hamburg, Germany

**Keywords:** Hairdressers, Wet work, Skin disease, Occupational disease, Prevention

## Abstract

**Background:**

Occupational skin disease caused by wet work is particularly common in employees in hairdressing salons. The objective of this paper was to determine the frequency of glove use for hair shampooing.

**Methods:**

Data on the usage of gloves for hair shampooing were collected by covert observations in four cross-sectional surveys of newly opened hairdressing salons located in Cologne. Measurements were conducted between 2009 and 2012. A team of five trained observers were involved in the measurements. As a second assessment method, salon owners of other newly opened salons from five districts of Germany were interviewed by telephone at three of the four measurement points. Trend analysis was performed with the Mantel-Haenszel test for trends and simple linear regression. Differences in proportions of glove use between the two assessment methods were compared by chi-squared tests.

**Results:**

In total, 435 hair shampoos were observed and 630 salon owners interviewed. Gloves were worn in 14 % of the observed hair shampoos. Proportions of glove use differed significantly according to assessment measurement. Proportions of glove use assessed by covert observation increased from 10.5 % to 18.5 % (p_trend_ = 0.044) whereas the proportions obtained by telephone interview decreased over the study period from 84 % to 76 % (p_trend_ = 0.037). No trend was found for the intensity of glove use (p_trend_ = 0.204).

**Conclusions:**

Gloves were worn in less than 20 % of hair shampoos. This rate is much lower than values reported from other written or verbal surveys. Future measures for skin protection in hairdressing salons should take this into account.

**Electronic supplementary material:**

The online version of this article (doi:10.1186/s12995-015-0089-y) contains supplementary material, which is available to authorized users.

## Background

Occupational hand eczema is one of the most prevalent occupational diseases (OD) in Europe [1]. The complications and consequences of occupational hand eczema include chronic severe eczema, prolonged sick leave, unemployment, and impaired quality of life [2]. In Germany, claims of occupational skin disease (OD No. 5101) accounted for around 34 % of all workers’ compensation claims in 2013 [3]. Around 4.5 % of all claims of occupational skin disease (OSD) were notified by hairdressers (Berufsgenossenschaft für Gesundheitsdienst und Wohlfahrtspflege, Statistical Department 2014, personal communication). However, more than 14.2 % of the recognised cases with OSD concerned hairdressers. Hairdressers have an increased risk of developing skin disease, as they are exposed to several hair dressing chemicals, such as perming agents, hair dyes and bleaches and work with irritants such as shampoos and water [4–6]. The duration of wet work in the hairdresser trade - measured by direct observation – ranges between 2 and 4 h per day [7, 8].

Preventive programs for employees in hairdressing salons were first introduced in Germany in 1992 and there has been a downward trend in suspected and recognised cases of OSD in hairdressers over the last 15 years (see also in Additional file 1). This seems to reflect improvements in working conditions in hairdressing salons, due to both new legislation [9, 10] and the introduction of measures for secondary and tertiary individual prevention [11]. Although the annual numbers of skin disease claims reported by hairdressers have decreased over the last decade, the annual incidence of new reports is still high - with approximately 4.0 to 5.0 cases per 1,000 full time employees (Berufsgenossenschaft für Gesundheitsdienst und Wohlfahrtspflege, Statistical Department 2014, personal communication). The annual workers’ compensation-related costs in Germany for medical treatment, professional rehabilitation and pensions for hairdressers with an OD No. 5101 were approximately 12 million Euro in 2000, but continuously decreased until 2007. Since 2007, the annual costs have stagnated at just under 8 million Euro (see Additional file 1). In this context, a campaign to prevent hand eczema in hairdressers was started in 2009 by the workers’ compensation board responsible for hairdressers in Germany - the Social Accident Insurance Institution for the Health and Welfare Services (Berufsgenossenschaft für Gesundheitsdienst und Wohlfahrtspflege, BGW).

### The prevention campaign for the hairdressing craft

The campaign *Live your dream* was directed towards employees in hairdressing salons. The campaign was designed to increase skin protection measures among hairdressers and ultimately reduce disability and workers’ compensation-related costs. The objective was to persuade employees in hairdressing salons to wear gloves for wet work. If gloves were worn more often for shampooing hair, this should lead to a reduction in the daily exposure to wet work and should correspond to increased skin protection.

The campaign’s claim was “with glamour, style and beautiful hands”. All the media and products used in the campaign were specifically designed to be appropriate to the target group. This was particularly the case for the central product in the campaign, the champagne coloured disposable nitrile glove, which was intended to be regarded as smart and trendy by the hairdressers and to encourage them to wear gloves for shampooing hair. Communication focussed on advertisements in the printed media, together with the Internet platform “Lebe deinen Traum” (*Live your dream*) and the social networks Facebook and Twitter. The Facebook page offered various links to skin protection measures by the BGW. An average of two contributions per day was posted in Facebook. The posts dealt with campaign topics, but also contained infotainment or referred to offers such as the Monday discount for ordering gloves. During the campaign, an online shop was operated, which sold skin care products including the campaign gloves.

The aim of our study was to quantify how often gloves were used by hairdressers for shampooing hair.

## Methods

### Study Design

Glove usage in the salons was assessed by two disparate methods - covert observations of hair shampoos and telephone interviews of salon owners. Cross-sectional surveys were conducted in hairdressing salons annually in August between 2010 and 2012 (Survey 2, 3 and 4). A baseline survey was administered prior to the start of the campaign in August 2009 (Survey 1).

### Participants and data collection

The data base consisted of German hairdressing salons which had been registered between January 2008 and June 2012 as newly opened salons in the address database of the workers’ compensation board (BGW). The inclusion criteria were that the salon had opened in the 12 months before the corresponding survey and was registered in one of the five district administrations of the BGW chosen as study regions (Munich, Dresden, Cologne, Magdeburg and Hamburg). In total, 10,264 newly opened salons were identified during the study period. Lists of salons were generated for each survey, consecutively numbered and transmitted to a research institute in the social sciences, that had been commissioned to perform the observations and to a market research institute, that had been commissioned to perform the telephone interviews. Random samples were drawn by the commissioned institutes, using random numbers generated by a random number generator. In order to compensate for drop-outs, the sample contained twice as many salons as the target number of observations, or five-fold the number of interviews. If no observation was possible in the salon or an interview was refused, this was replaced by the next entry in the list of random numbers.

### Covert observation

The observation of shampooing was based on the methods of participant and non-participant observation [12]. In the participating variant, the researcher became part of the situation being studied, e.g. had his or her hair shampooed; in the non-participating variant, the researcher attempted to observe whether the hairdressers wore gloves for shampooing the hair of other customers. The observations were covert. The salon owner was not informed about the observation, in order to avoid the effect of social desirability. The trained observer team consisted of four females and one male person. The participating and non-participating observations were performed in a standard manner (including for example making an appointment or leafing through hairdressing magazines). Each observation was documented retrospectively with the help of a structured observation form. The documentation form contains information on the date and time of the observation, as well as the number and gender of the customers and employees, the number of observed hair shampoos and whether these were performed in gloves. The endpoint of the analysis was, whether gloves were used for shampooing during the observation, with the options yes and no. The distinction between the participating and non-participating variants was dispensed with. If no shampooing had been performed within a limited time frame of 20 min, the observation was coded as unsuccessful.

A pre-test was performed in 10 salons - five each from an urban area and a rural area of Cologne. It became apparent that too much time was needed for observations in rural areas - long drives, few customers or long waiting times for non-participating observations. For this reason, a sample of salons located in the greater Cologne area was selected. The address database for observations included a total of 3,019 salons. A random sample of 749 salons was visited to conduct the observation of shampoos (32 % out of 2,339 salons with verified addresses; 241 in survey 1, 163 in survey 2, 88 in survey 3 and 257 in survey 4). Routes were arranged for the observation team along the addresses in the random sample. Observation was successful in every second salon (385 out of 749).

### Telephone survey

Telephone interviews were restricted to the owners, as they bear the responsibility for implementing the health and safety regulations in their salons. A computer-assisted telephone interview questionnaire was used. The questionnaire comprised two main topics; the first topic contained questions on implementation of skin protection measures in the salon, glove use during daily work (including hair shampooing) and reasons for non-use of gloves for hair shampooing. The second topic contained questions on the perception of the campaign and the use of the campaign materials; these results will be reported elsewhere [13]. Glove use was assessed with two questions: The first question was “Are gloves used for shampooing in your salon?” with the options yes and no; the second question was “How often do the employees in your salon use gloves for shampooing?” with the options never, rarely, frequently and always.

Data collection by telephone interview was limited to surveys 1, 2 and 4. The address database for the telephone surveys included a total of 7,578 salons. A random sample of 2,958 salons with verified phone numbers was used. Contact by telephone interview was made with 1,820 salons (433 from survey 1, 575 from survey 2, and 812 from survey 4).

Compliance with the rules on data protection was confirmed by the data protection manager of the BGW.

#### Statistical analysis

Data analysis was based on the number of hair shampoos that had been observed and — in the case of the telephone survey — on the number of salon owners who had agreed to participate in the interview. Data from each survey were used as cross-sectional samples. The Mantel-Haenszel test for trend and linear regression were used for the analysis of trends in the proportion of glove use across the study period. Differences in proportions between the two assessment measurements were compared by the Chi-square test. A p value of less than 0.05 was considered as statistically significant. The statistical analyses were performed using IBM SPSS version 20 and MedCalc software.

## Results

A total of 435 hair shampoos were observed and a total of 630 salon owners (35 %) agreed to be interviewed. Gloves were worn in 14.0 % of the observed hair shampoos (Table 1). The proportion of glove use increased from 10.5 % in survey 1 to 18.5 % in survey 4, with a mean increase of 2.7 percentage points per year (*p*_*trend*_ = 0.044). According to the interviews, protective gloves were available in the salons of nearly all interviewed salons owners (Table 1). However, gloves were used for shampooing in only 80.3 % of the salons using protective gloves generally. The proportion of glove use decreased from 84.4 % to 76.2 % over the study period with a mean decrease of 2.6 percentage points per year (*p*_*trend*_ = 0.037).

**Table 1:** Proportions of glove use for hair shampooing in hairdressing salons; separated by methods of assessment

		Observation of shampoos			Telephone interview of salon owners				Comparison of proportion for glove use for hair shampooing	
		Number of salons	Glove Usage		Glove Usage					
					Generally		For shampoos			
Survey	Data collection	N	N	%^a^	N	%^a^	N	%^b^	*χ* ^2^	P
1	August 2009	172	18	10.5	218	99.1	184	84.4	207.564	<.001
2	August 2010	69	9	13.0	199	99.5	159	79.9	95.068	<.001
3	August 2011	75	12	16.0	-	-	-	-	-	-
4	August 2012	119	22	18.5	198	94.3	151	76.2	97.763	<.001
Total		435	61	14.0	615	98.0	494	80.3	446.824	<.001

^a^Relative to number of salons (observed/interviewed)

^b^Relative to number of salons using gloves generally

Rates for the intensity of glove use showed that gloves were worn in 39 % of hair shampoos always (Table 2). The figure is similarly high for frequent use of glove. In around 25 % of shampoos gloves were worn only rarely or never. Rates for the intensity of glove use did not change statistically significantly across the three surveys (*p*_*trend*_ = 0.204).

**Table 2:** Intensity of use of gloves when shampooing hair according to telephone survey (N = 603)

	Always		Frequently		Rarely/never	
Survey	N	%	N	%	N	%
1	87	40.6	86	40.2	41	19.2
2	71	36.4	72	36.9	52	26.7
4	77	39.7	63	32.5	54	27.8
Total	235	39.0	221	36.6	147	24.4

The rates of glove use provided by the salon owners were much higher for all surveys than the rates determined by observation - by a factor of about 6 (Table 1). However, if the comparison is restricted to hairdressers who always use gloves for shampooing (39 %, Table 2), this gap is narrowed to a factor of 3.

## Discussion

The campaign *Live your dream* was developed to make hairdressers more aware of the issue of skin protection. The principle objective of the campaign was to reduce the high exposure to wet work by increasing the use of gloves for shampooing hair. The campaign focused on enhancing the attractiveness of glove use for hairdressers so that this desirable procedure became routine for them.

There is currently no gold standard for assessing wet work that is stressful for the skin during the working day [8]. In epidemiological studies, video cameras or hand-held computers are often used to record occupational exposure. However, neither of these can be used without the knowledge of the observed employees and it is conceivable that they may change their behaviour.

According to the assessment method of covert observation, the overall rate of glove use for hair shampoos is around 14 %. To our knowledge, this is the first study to apply the method of covert observations in hairdressing salons in order to assess the usage of gloves for hair shampooing. Therefore no directly comparable figures exist. Rates of glove use for shampooing published by other studies vary between 6 % and 76 % [14–18]. Our rate of 14 % lies at the lower end of this broad range. One explanation for the discrepancy may be that the published figures for glove use were predominantly collected by questioning hairdressers. The rates of glove usage provided by the salon owners fit well with the range of the published data. The high rates suggest that the answers were influenced by the social desirability of the responses, as the salon owners were aware that the survey had been commissioned by the workers’ compensation company that is responsible for hairdressers. This may indicate that cognitive dissonance is given, that the salon owners possessed the requisite knowledge, but that they often failed to implement it. There is no conclusive explanation for the decrease in glove use over time assessed by interview.

As shown by the observations, there was a positive trend for glove use for shampooing across the four surveys. Although measurement points cover the duration of the prevention campaign, it is unclear whether this improvement was caused by the campaign’s activities.

### Strength and limitations

The method of covert observation has been applied for the first time to a large group of hairdressing salons, in order to assess the frequency of glove use. The method of covert observation gives the opportunity to document workplace behaviour in hairdressing salons without the employees being aware and acting in the sense of social desirability. On the other hand, the observations were very tedious, with respect to both time and costs. The limitation to one observation per salon can lead to bias.

The present study design cannot be used for an evaluation of the effectiveness of the prevention campaign *Live your dream*, as no control group was included and no cohort of salons with repeated pre-/post measurements beyond the end of the campaign was provided. As it is not known whether the hairdressers in the campaign salons had changed their intentions and accepted glove wearing for shampooing as their new behaviour, no association can be established between the campaign’s activities and proportions of glove usage assessed by our study.

## Conclusions

The rate of glove use for hair shampoos is less than 20 % and is thus considerably lower than reported in earlier studies based on data provided by questionnaire-based surveys.

## Additional file

Additional file 1:
**Figure “Occupational dermatitis in German hairdressers. Number of suspected and recognised cases and compensation payments (1999-2014; BGW data)”.** (XLSX 14 kb)
